# Editorial: Mechanisms and strategies to overcome antibiotic resistance in gastrointestinal pathogens

**DOI:** 10.3389/frabi.2025.1706166

**Published:** 2025-10-01

**Authors:** Tales Fernando da Silva, Bartłomiej Grygorcewicz

**Affiliations:** ^1^ Physiology and Biophysics Department, Institute of Biological Sciences, Federal University of Minas Gerais, Belo Horizonte, Brazil; ^2^ Department of Genomics and Forensic Genetics, Pomeranian Medical University in Szczecin, Szczecin, Poland

**Keywords:** antibiotic resistance, gastrointestinal pathogens, *Helicobacter pylori*, traditional Chinese medicine (TCM), non-antibiotic therapies

Antibiotic resistance in gastrointestinal (GI) pathogens represents one of the most pressing global health challenges of our time. Infections caused by *Helicobacter pylori*, *Salmonella*, *Escherichia coli*, and *Campylobacter* account for substantial morbidity and mortality worldwide, and their treatment is increasingly compromised by resistance to first-line antibiotics. This Research Topic, Mechanisms and Strategies to Overcome Antibiotic Resistance in Gastrointestinal Pathogens, brings together studies that illuminate the molecular and clinical dimensions of resistance, while exploring alternative therapeutic avenues ranging from traditional medicine to probiotics and bacteriophages. Collectively, these contributions underscore the urgent need for innovative diagnostic tools, integrative treatment strategies, and translational research to confront the multifactorial nature of resistance.

## Resistance surveillance: defining the scope of the problem

Accurate mapping of resistance prevalence and mechanisms remains a cornerstone in the management of GI infections. In this Research Topic, Chen et al. present data from Dongying, on China’s eastern coast, where *H. pylori* infection rates reached nearly 25%, with alarming resistance rates exceeding 60% for clarithromycin and 46% for levofloxacin (Chen et al.). By combining non-invasive string tests with qPCR, they demonstrate a feasible approach for real-time resistance profiling, reducing the need for invasive biopsies. This methodological innovation is not only suitable for *H. pylori*, but also exemplifies how diagnostics can be refined for broader GI pathogens.

Such findings align with global surveillance studies reporting increasing resistance in *Campylobacter* and *Salmonella*, driven by overuse of antibiotics in both healthcare and agriculture (WHO, 2023). At the molecular level, resistance arises from mutations in ribosomal RNA genes, DNA gyrase alterations, efflux pump overexpression, and biofilm formation (Lamberte and van Schaik, 2022). Integration of molecular diagnostics and next-generation sequencing into clinical practice will therefore be vital for precision treatment and prevention of further resistance spread.

## Harnessing traditional medicine in the fight against resistance

As antibiotic efficacy wanes, attention has turned to non-conventional therapies. Two studies in this Topic highlight the promise of traditional Chinese medicine (TCM) in combating *H. pylori*.

Ou et al. evaluated the Helile formula, derived from the “Taiping Shenghuifang,” revealing its capacity to disrupt *H. pylori* structure, inhibit virulence gene expression, and modulate host inflammation (Ou et al.). Importantly, multi-omics profiling indicated shifts in metabolites and gut microbiota, suggesting a systemic impact beyond bacterial killing.

Complementarily, Yang et al. conducted a multicenter randomized trial assessing the Jinghua Weikang capsule (JWC) combined with rescue therapies in patients with refractory *H. pylori* infection (Yang et al.). JWC-containing regimens demonstrated eradication rates comparable to bismuth quadruple therapy, with fewer adverse events and improved symptom relief. Notably, prolonged JWC administration was non-inferior to standard rescue therapy, reinforcing the idea that herbal formulations may enhance both efficacy and tolerability.

These findings reflect a broader trend where herbal medicines and natural products are scientifically validated for antimicrobial properties (Newman and Cragg, 2020). They highlight how integrating traditional formulations with rigorous modern methods can provide credible alternatives in resistant GI infections.

## Expanding the therapeutic horizon: non-antibiotic strategies

The search for antibiotic-sparing therapies extends beyond TCM. In their comprehensive review, Oliveira et al. highlight the therapeutic potential of probiotics, natural compounds, and bacteriophages in managing multidrug-resistant GI infections (Oliveira et al.).

Probiotics, such as *Lactobacillus* and *Bifidobacterium* species, exert effects through competitive exclusion of pathogens, enhancement of mucosal barriers, and modulation of immune responses (da Silva et al., 2024). Clinical trials support their role in reducing recurrence of *Clostridioides difficile* infections and alleviating side effects of *H. pylori* eradication therapy (Esmaeilinezhad et al., 2025). Natural compounds—including polyphenols, flavonoids, and essential oils—display antimicrobial effects through diverse mechanisms, from membrane disruption to quorum sensing inhibition (Lewis et al., 2024). Bacteriophages, with their pathogen-specific action, are being explored as precision antimicrobials against resistant enteric pathogens (Olawade et al., 2024).

The review also underscores the potential of combining these approaches and tailoring interventions to patient-specific microbiome profiles, aligning with the principles of personalized medicine (Terra et al., 2024).

## Toward integrated solutions

Taken together, the contributions in this Research Topic highlight both the scale of the resistance crisis and the innovative directions emerging to address it. From surveillance studies providing region-specific prevalence data, to clinical trials demonstrating the efficacy of TCM-based regimens, and reviews synthesizing the state of the art in non-antibiotic therapies, the picture that emerges is one of cautious optimism.

To truly overcome antibiotic resistance in GI pathogens, several priorities emerge:

- Enhanced diagnostics: Implementation of rapid, minimally invasive methods for pathogen detection and resistance profiling.
- Mechanistic insights: Continued molecular characterization of resistance pathways, supported by pangenomic and in silico analyses.
- Therapeutic diversification: Integration of herbal medicines, probiotics, natural compounds, and phages into mainstream care, supported by high-quality trials.
- Stewardship and regulation: Strengthened frameworks to ensure safe, standardized deployment of non-antibiotic therapies.
- One Health perspective: Recognition that resistant pathogens circulate between humans, animals, and the environment, necessitating cross-sectoral interventions.

This Research Topic underscores the necessity of a multidimensional approach. While antibiotics remain indispensable, their limitations in the face of resistance demand complementary strategies. By integrating precision diagnostics, alternative therapeutics, and global stewardship, we move closer to sustainable solutions against resistant GI infections. The works collected here not only deepen our understanding of resistance mechanisms but also chart practical pathways for innovation. In doing so, they contribute to the collective goal of safeguarding effective treatments for future generations.
